# Seasonal and Gender Differences in Presence of *Rickettsia felis* and Blood meals Provide Additional Evidence of a Vector Role for Mosquitoes

**DOI:** 10.1155/2019/8543460

**Published:** 2019-04-10

**Authors:** Jilei Zhang, Guangwu Lu, Patrick John Kelly, Chengming Wang

**Affiliations:** ^1^College of Veterinary Medicine, Yangzhou University, Yangzhou, Jiangsu, China; ^2^Division of Gastroenterology and Hepatology, Department of Medicine, University of Illinois at Chicago, Chicago, Illinois, USA; ^3^Ross University School of Veterinary Medicine, Basseterre, Saint Kitts and Nevis; ^4^College of Veterinary Medicine, Auburn University, Auburn, AL, USA

## Abstract

*Rickettsia felis* belongs to spotted fever group *Rickettsia* and is an emerging human pathogen most commonly transmitted by a range of fleas and ticks. While recent evidence has suggested mosquitoes are infected with *R. felis*, there is little information about the role of mosquitoes in the organism's transmission. In this study, around 100 mosquitoes were collected monthly between 2013 and 2014 from the same residential dwelling at Yangzhou, China. The collected mosquitoes were identified for their species and gender, followed by *gltA*-based PCR and hydroxymethylbilane synthase-based PCR to determine the prevalence of *Rickettsia* and blood meal. Three mosquito species (*Culex pipiens*: 76%, 996/1,304; *C. tritaeniorhynchus*: 17%, 216/1,304; *Aedes albopictus*: 7%, 92/1,304) were identified. For 1,088 female mosquitoes, 31% of them (*n*=336) were positive for blood meal and 7% (*n*=77) carried *R. felis* DNA. In a strong contrast, none of the 216 male mosquitoes were positive for blood meal but two males were positive for *Rickettsia*. Interestingly, 63% of *R. felis-*positive mosquitoes (50/79) were negative for blood meal, being significantly higher than 37% of mosquitoes and being positive for both *R. felis* and blood meal (*P*=0.008). Furthermore, we compared the prevalence of *Rickettsia* and blood meal in the mosquitoes collected in the months with temperature below and above 23°C, the minimum temperature required for mosquito egg hatching. Mosquitoes captured in the months below 23°C showed significant higher positivity of *R. felis*(71/936, 7.6% vs. 8/368, 2.2%; *P*=0.002) and blood meal (294/936, 31.4% vs. 36/368, 9.8%; *P* < 10^−4^) than in the months above 23°C. Collectively, the seasonal and gender differences of *R. felis* and blood meal in mosquitoes add to the existing evidence, supporting a potential vector role of mosquitoes in the transmission of *R. felis*. Studies with a *R. felis* infection model covering the full life cycle of mosquitoes is necessary to unambiguously prove the transstadial and transovarial transmission of *R. felis* in mosquitoes.

## 1. Introduction

*Rickettsia felis* is an obligate intracellular Gram-negative bacterium belonging to the family *Rickettsiaceae* [1] and the agent of flea-borne spotted fever in people [2]. Except the primary vector of fleas, *R. felis* was detected in mosquitoes by a growing number of recent reports, paralleling the increasing implication of *R. felis* as a human pathogen [3–9]. Additionally, it was suggested that *Rickettsia* may be maintained in mosquitoes through both transstadial and transovarial transmission [4, 7, 8]. *R. felis* can grow in some mosquito cell lines, such as *Aedes albopictus* and *Anopheles gambiae* [10], indicating the possibility of the transmission and evolution of *Rickettsia* in the mosquito.

Mosquitoes are cosmopolitan in every land region with huge numbers except for Antarctica and a few islands [11]. Feeding on blood is a behavioral trait of female mosquitoes that allows them to obtain the nutrients necessary for reproduction [12]. Male mosquitoes mainly feed on nectar and plant juices, but not blood meal. However, the number and abundance of mosquito species and the blood-sucking activities are variable due to the vectors of climatic circumstances, animals, and human activities. Investigation on the seasonal differences of *R. felis* and blood meal in female and male mosquitoes will lead to a better understanding of this organism's transmission.

Here, molecular approaches were used to determine the positivity of *Rickettsia* and blood meal of the mosquitoes captured from the same residential dwelling between September 2013 and August 2014. The findings of this study were described as below.

## 2. Materials and Methods

### 2.1. Sample Collection

**Figure 1:**
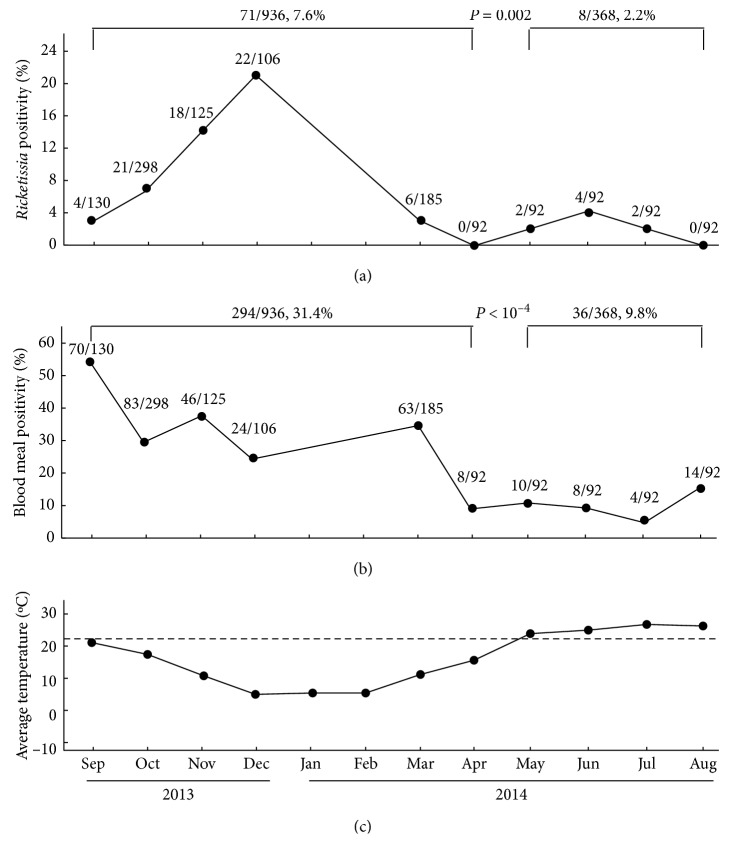
Significant higher *R. felis* and blood meal positivity in mosquitoes collected when the temperature was lower than 23°C than above 23°C. Mosquitoes collected in months with average temperatures below 23°C showed significant higher positivity of *R. felis* ((a); 71/936, 7.6% vs. 8/368, 2.2%; *P*=0.002) and blood meal ((b); 294/936, 31.4% vs. 36/368, 9.8%; *P* < 10^−4^) than collected between May and August with above 23°C temperature (c).

Between September 2013 and August 2014, mosquitoes (*n*=1, 304) were captured monthly with hand nets in the same residential dwelling of Yangzhou, Jiangsu, China (Table 1, Figure 1). The collection of mosquitoes was not performed in January and February of 2014 when the university was closed for the holiday. After washing in PBS to exclude the possibility of environmental contamination with *R. felis*, the species and gender of the collected mosquitoes were identified using standard morphological criteria [15] and PCR before putting into 400 *μ*l (individual)/600 *μ*l (pool) of RNA/DNA Stabilization Reagent (Roche Molecular Biochemicals, Indianapolis) and then stored at −80°C freezer until DNA extraction as described below. The average monthly temperatures in the Yangzhou were obtained from the local weather station.

### 2.2. DNA Extraction

After thawing and triturating with the shaker (Precellys 24 sysis and homogenization, France) at 5800 × rpm, 15 s × 2 (times), and 20 s (interrupt) at room temperature, mosquitoes were used for DNA extraction with QIAamp DNA Mini Kit (QIAGEN, Valencia, USA) according to the manufacturer's descriptions.Then, the extracted DNAs were kept in −80°C freezer until performance of PCR assay detection.

### 2.3. PCR Assays

HMBS gene (hydroxymethylbilane synthase gene) is a single-copy gene of the heme synthesis pathway in mammalians but not plants and prokaryotes (Table 1). The HMBS-basedFRET-qPCR targeting for a 286 bp amplicon was used for blood meal detection in mosquitoes as described before [4, 13]. The HMBS-basedFRET-qPCR was proved to be highly sensitive and specific and was confirmed to detect 13 mammalian species of blood meals in mosquitoes [13].

The *gltA*-based FRET-qPCR targeting for a 170 bp amplicon of *Rickettsia* spp. and nested-PCR targeting for 446 bp and 353 bp amplicon *gltA* gene were used in this study to detect *Rickettsia* infections as described before [4, 5]. The *gltA* gene encodes one type of citrate synthase which plays a key role in energy production and providing biosynthetic precursors. The *gltA*-based PCRs were established with high sensitivity and specificity and applied in variety of samples including mosquitoes [4, 5].

The species of mosquitoes were also verified by the PCR assay with amplicon of 710 bp cytochrome c oxidase subunit 1 gene (*CO1* gene) as described before [14, 16]. *CO1* gene is one of three mitochondrial DNA-encoded subunits of cytochrome c oxidase which is a key enzyme in aerobic metabolism. It is proposed as the DNA barcoding system for animal life [17] and had been used as the DNA barcodes for the common mosquito species in China [16].

### 2.4. Statistical Analysis

The chi-squared test (Statistica, StatSoft, Tulsa, USA) was used to compare the prevalence of *Rickettsia* and blood meal between different groups of mosquitoes. *P* < 0.05 was considered significantly different.

## 3. Results and Discussion

According to the morphological criteria and PCR followed by DNA sequencing, three mosquito species were identified in this study, including *Culex pipiens* (76%, 996/1,304), *C. tritaeniorhynchus* (17%, 216/1,304), and *Aedes albopictus* (7%, 92/1,304), in both female (83%, 1088/1,304) and male (17%, 216/1,304) (Table 1, Figure 1).

*R. felis* was detected in all the months with mosquito samples except in April and August of 2014 (6%, 79/1,304). We found that about one-third of the female mosquitoes (31%, 336/1,088) had taken a blood meal while 7% (77/1,088) carried *R. felis* DNA. In strong contrast, none of the 216 male mosquitoes were positive for a blood meal but two were *R. felis* positive. The absence of a blood meal in the males was anticipated as males do not feed on animals but obtain their nutrition from plants. Detection of *R. felis* DNA in male mosquitoes, however, suggests vertical (transovarial) transmission of *R. felis* in mosquitoes such that the organism could be maintained in the population without mosquitoes having to feed on rickettsemic hosts.

In this study, 79 *R. felis*-positive mosquitoes were identified. For these *Rickettsia*-positive samples, 63% of them (50/79) were free of a blood meal, being significantly higher than 37% being also positive for blood meal (29/79) (*P* = 0.008) (Table 2, Figure 1). This further stipulates the possibility of vertical transmission of this organism in mosquitoes.

Yangzhou, located in the east of China with the coordinates of 32°24′N, 119°25′E, has a subtropical monsoon climate with humid changeable wind, longer winter (4 months) and summer (3 months), and shorter springs and autumns (2 months each). The annual average temperature is 15°C with the highest in July (40°C) and lowest in January (−15°C). The average atmosphere temperature for each of the four months (May, June, July, and August) of 2014 was above 23°C, the minimum temperature required for mosquito egg hatching [18, 19]. Species richness and number abundance of the mosquito were generally higher during the summer and fall [20]; for instance, *C. pipiens* appear around May, and the density slowly increased until a seasonal maximum in July-August [21] or sometimes later in September [22], which is confirmed by our findings in this study. The temperature is critical to key life-fitness parameter of the stages of many insect species [23], including mosquitoes. The development thermal threshold (23°C) (Figure 1(c), dash line) is the temperature below which immature stages would stop developing [18, 19]. The data in this study indicated that the positivity of *R. felis* (2%, 8/368 vs. 8%, 71/936, *P*=0.002) and blood meal (10%, 36/368 vs. 31%, 294/936, *P* < 10^−4^) in these four months above 23°C was significantly lower than those months below 23°C (Figure 1). Mosquitoes captured in the months without the possibility of mosquito egg hatching had significantly higher prevalence of *R. felis* than those in the months with constant egg hatching. This difference may add to the evidence suggesting the transstadial and transovarial transmission of *R. felis* in mosquitoes. In order to unambiguously prove the transstadial and transovarial transmission of this organism in mosquitoes, further studies with a *R. felis* infection model covering each life stage of mosquitoes is necessary.

## 4. Conclusions

In conclusion, the seasonal and gender differences of *R. felis* and blood meal in mosquitoes suggest the possible transstadial and transovarial transmission of *R. felis* in mosquitoes. Studies with the *R. felis* infection model within the full life cycle of mosquito are necessary to unambiguously prove the potential vector role of mosquitoes in transmission of this organism.

## Figures and Tables

**Table 1:** Primers and probes used in the PCR reactions in this study.

PCR	Primer/probe nucleotides	Amplicon	Reference
HMBS FRET-qPCR	Forward primer: 5′-tacctgactggaggagtctggagtct-3′	286 bp	[13]
	Downstream primer: 5′-gccaggctgatgcccaggttct-3′		
	Anchor probe: 5′-agcahgaagatggyccwga-6-FAM-3′		
	Reporter probe: 5′-LCRed640-gatgayccacagctggtrg-phos-3′		

*Rickettsia gltA* FRET-qPCR	Up primer: 5′-ttrcaaatagcaatagaacttgaagct-3′	170 bp	[5]
	Reverse primer: 5′-agcaagaaccgtaggctggat-3′		
	Anchor probe: 5′-atcgctcttaaagatgaatattttattgag-6-FAM-3′		
	Reporter probe: 5′-LCRed640-gaaaattatatccaaatgttgatttttattc-phos-3′		

*Rickettsia gltA* nested-PCR	Out-up primer: 5′-agtaaatccaataataaaaaatgckcttaata-3′	446 bp	[5]
	Out-down primer: 5′-cttaaagatgaatattttattgagagaaaat-3′		
	In-up primer: 5′atgagcagaatgcttctacttcaaca-3′	353 bp	
	In-down primer: 5′-ttrcaaatagcaatagaacttgaagct-3′		

Mosquito *COI* PCR	LCO1490: 5′-ggtcaacaaatcataaagatattgg-3′	710 bp	[14]
	HC02198: 5′-taaacttcagggtgaccaaaaaatca-3′		

**Table 2:** Prevalence of *R. felis* and blood meal in mosquitoes in this study.

Date	Sample #	Gender and species	*R. felis*+^*∗*^	Blood meal+	*R. felis* + blood meal+	*R. felis* + blood meal−
Sep. 2013	130	70 ♀ *Culex pipiens*	3%, 2/70	54%, 38/70	100%, 2/2	0%, 0/2
		60 ♀ *C. tritaeniorhynchus*	3%, 2/60	55%, 33/60	100%, 2/2	0%, 0/2

Oct. 2013	298	164 ♀ *C. pipiens*	7%, 12/164	29%, 48/164	42%, 5/12	58%, 7/12
		134 ♀ *C. tritaeniorhynchus*	7%, 9/134	28%, 37/134	11%, 1/9	89%, 8/9

Nov. 2013	125	103 ♀ *C. pipiens*	15%, 15/103	38%, 39/103	47%, 7/15	53%, 8/15
		22 ♀ *C. tritaeniorhynchus*	14%, 3/22	36%, 8/22	67%, 2/3	33%, 1/3

Dec. 2013	106	106 ♀ *C. pipiens*	21%, 22/106	23%, 24/106	27, 6/22	73%, 16/22

Mar. 2014	185	185 ♀ *C. pipiens*	3%, 6/185	34%, 63/185	33%, 2/6	67%, 4/6

April 2014	92	48 ♀ *C. pipiens*	0%, 0/48	17%, 8/48	0%, 0/0	0%, 0/0
		44 ♂ *C. pipiens*	0%, 0/44	0%, 0/44	0%, 0/0	0%, 0/0

May 2014	92	52 ♀ *C. pipiens*	4%, 2/52	21%, 11/52	50%, 1/2	50%, 1/2
		40 ♂ *C. pipiens*	0%, 0/40	0%, 0/40	0%, 0/0	0%, 0/0

June 2014	92	52 ♀ *C. pipiens*	4%, 2/52	17%, 9/52	50%, 1/2	50%, 1/2
		40 ♂ *C. pipiens*	5%, 2/40	0%, 0/40	0%, 0/0	100%, 2/2

July 2014	92	40 ♀ *C. pipiens*	5%, 2/40	10%, 4/40	0%, 0/0	100%, 2/2
		52 ♂ *C. pipiens*	0%, 0/52	0%, 0/52	0%, 0/0	0%, 0/0

Aug. 2014	92	52 ♀ *Aedes albopictus*	0%, 0/52	27%, 14/52	0%, 0/0	0%, 0/0
		40 ♂ *Aedes albopictus*	0%, 0/40	0%, 0/40	0%, 0/0	0%, 0/0

Total	1304	996 *C. pipiens*	6%	31%	37%, 29/79	63%, 50/79
		216 *C. tritaeniorhynchus*	79/1,304	336/1,088		
		92 *Aedes albopictus*				

^*∗*^* Rickettsia* positive/negative, blood meal positive/negative.

## Data Availability

The data used to support the findings of this study are included within the article.
